# Research progress on classical traditional chinese medicine formula xiaoyaosan in the treatment of depression

**DOI:** 10.3389/fphar.2022.925514

**Published:** 2022-08-04

**Authors:** Jianbei Chen, Chaofang Lei, Xiaojuan Li, Qian Wu, Chenyue Liu, Qingyu Ma, Jiaxu Chen

**Affiliations:** ^1^ School of Traditional Chinese Medicine, Beijing University of Chinese Medicine, Beijing, China; ^2^ Formula-pattern Research Center, School of Traditional Chinese Medicine, Jinan University, Guangzhou, China; ^3^ Institute of Chinese Materia Medica, China Academy of Chinese Medical Sciences, Beijing, China

**Keywords:** xiaoyaosan, TCM, depression, clinical application, pharmacological mechanism, research progress, review

## Abstract

Depression is an emotional disorder that is problematic in psychiatry owing to its unclear etiology and unknown pathogenesis. Traditional Chinese medicine formulations such as Xiaoyaosan have been widely used throughout history to treat depression. In this review, we have focused on recent evidences elucidating the links between Xiaoyaosan and the treatment of depression. Data from animal and clinical studies, focusing on the pharmacological mechanisms, clinical applications, and effective materials that form the basis for the treatment of depression are presented and discussed. We found that the antidepressant effects of Xiaoyaosan are related to the effects of monoamine neurotransmitters, regulation of the hypothalamic-pituitary-adrenal axis, neuroplasticity, synaptic plasticity, inflammatory response, neuroprotection, brain-gut axis, regulation of intestinal microbiota, oxidative stress, and autophagy for reducing neuronal apoptosis. This review highlights the current evidence supporting the use of Xiaoyaosan as an antidepressant and provides an overview of the potential mechanisms involved.

## 1 Introduction

Depression is a common emotion-related psychiatric disease encountered in clinical practice, with a global prevalence of 4.4–5%. Its primary clinical characteristics include lasting depression, impaired thinking and cognitive function, and decreased activity, in addition self-mutilation, suicide, and other associated behaviors can also occur in more severe cases (Ferrari et al., 2013; Smith 2014). According to the Global Burden of Disease Study 2013, major depressive disorder (MDD) is the second leading cause of years lived with disability (Vos et al., 2015). The population-attributable risk for depression and all causes of death is 12.7% and for depression and suicide it reaches 11.2% (Walker et al., 2015). According to the World Health Organization (WHO), depression has become one of the main global causes of disability, and it is expected to become a main disease burden and a major cause for increased medical expenses by 2030 (Mathers 2008; Yao et al., 2015). Due to the complex and diversified clinical manifestations of depression, it is difficult to predict the course and prognosis of this disease. Furthermore, patient responses to treatment are also variable, making diagnosis and treatment even more complex. Modern medical research has demonstrated that genetic factors can determine the susceptibility to depression, while environmental factors can be triggers. The occurrence and development of depression is thus closely related to physiological state, psychological state, and social environment of an individual (Figueiredo et al., 2015; Raič 2017). Although the pathogenesis of depression remains unknown, current medical theories consider the effects of monoamine neurotransmitters, immunodeficiency, hypothalamus-pituitary-adrenal (HPA) axis system activation, the neuroplasticity/synaptoplasticity hypothesis, and the intestinal microbiota hypotheses. In diagnosed patients, the concentration of monoamine neurotransmitters such as 5-hydroxytryptamine (5-HT), norepinephrine (NE), and dopamine (DA) are decreased, disrupting normal neuronal activities. Furthermore, an increase of interleukin-6 (IL-6), IL-1β, and tumor necrosis factor-alpha (TNF-α) results in immune system imbalance, and the over-activation of the HPA axis leads to neuroendocrine system dysfunction. Nerve cell plasticity is changed by neuronal injury and decreased brain-derived neurotrophic factor (BDNF) content, and dysregulation of the intestinal flora composition and function may result in metabolic abnormalities that lead to depression (Felger, 2016; Grace, 2016). These theories have been crucial for guiding the clinical treatment of depression. However, depression is currently treated in western medicine by using oral antidepressants, with limited response rate and delayed onset of efficacy due to their long therapeutic cycles, notable side effects, high price, and poor compliance (Health Quality, 2017). These drugs all act on specific parts of the brain and modulate brain function accordingly. However, most of these drugs can be severely toxic to patients, and chronic treatment of depression can also lead to structural changes in certain parts of the brain (Siddiqi et al., 2022). An increasing number of studies has demonstrated the outstanding efficacy of traditional Chinese medicine (TCM) and its compound preparations in the treatment of depression, with benefits such as high remission rates, long-lasting antidepressant effects, and fewer adverse reactions (Liu et al., 2015; Wu et al., 2015). TCM could thus be used to source a prospective alternative therapy for the treatment of depression. Phytochemicals are a safe, cost-effective, and highly effective antidepressant. A growing number of preclinical and clinical studies have revealed a complex array of psychoactive substances in herbal medicines that are beneficial in the treatment of depression (Sarris et al., 2011). Plant molecules isolated from medicinal plants produce antidepressant effects by modulating the levels of neurotransmitters such as dopamine, serotonin, and norepinephrine in different parts of the brain. The antidepressant mechanism of action of phytochemicals also includes negative regulation of monoamine oxidase and acetylcholinesterase activity and prevention of hyperactivity of the HPA axis. It also has strong antioxidant and anti-inflammatory potential, which provide synergistic effects for its antidepressant function (Kumar and Sharma 2022). Some herbal remedies have been approved by regulatory agencies to treat mental illness. For example, the Brazilian Health Regulatory Agency (Anvisa) has approved certain products derived from passionflower, valerian, cohosh, and Piper methysticum for the treatment of anxiety or depression. The European Medicines Agency has listed *Hypericum perforatum* (St. John’s Wort), *Melissa officinalis* L. (Melissa leaf), and *V. officinalis* L. (Valerian root) as plants approved for the treatment of stress and mood disorders (Fathinezhad et al., 2019). The State Drug Administration of China approved the traditional Chinese medicines Jieyuchufan Capsule, Morinda Oligosaccharide Capsule, and Shuganjieyu Capsule to treat mild to moderate depression. In addition, Xiaoyaosan is another popular Chinese herbal formula and one of the most widely used Chinese herbal formulas for the treatment of depression in China. This means that, with the continuous development of traditional Chinese medicine, the treatment of depression using plants has gradually been more widely recognized in the world, and the use of effective natural medicines instead of chemicals has become a new trend in the development of international medicine.

In TCM, depression is categorized as an “emotional disease” and it is described as “insomnia,” “lily disease,” and “depression syndrome”. Research on the formulations of TCMs is ongoing. Studies have found that some TCM formulations used to treat depression have multiple targets, low toxicity, and strong efficacy but their molecular mechanisms have not yet been fully clarified (Gao et al., 2013). Xiaoyaosan (XYS) which was first recorded in the *Taiping Huimin Heji Jufang* (Pharmacopeia of the Welfare Dispensary Bureau) by the Song Dynasty (960–1127 AD) is a classic Chinese medicinal compound. Its functions include reconciling the liver and spleen, soothing the liver, invigorating the spleen, nourishing the blood, and relieving depression. It is mainly used to treat liver stagnation, blood deficiency, and spleen weakness (Zhang et al., 2012; Du et al., 2014). XYS has been utilized in clinical practice in China for over 1,000 years, and it has often been used to treat a variety of conditions including depression, functional dyspepsia, chronic gastritis, and perimenopausal syndrome. Statistics on the frequency of symptoms treated using TCM show that the most common conditions related to depression were liver Qi stagnation and spleen deficiency. XYS is prepared using a traditional recipe that requires eight commonly used Chinese herbs [Chaihu (*Radix Bupleuri*), Danggui (*Radix Angelicae Sinensis*), Baishao (*Radix Paeoniae Alba*), Baizhu (*Rhizoma Atractylodis Macrocephalae*), Fuling (*Poria*), Bohe (*Herba Menthae Haplocalyx*), Shengjiang (*Rhizoma Zingiberis*), and Gancao (*Radix Glycyrrhizae*)], which have been used for centuries to treat mental illnesses, including depression (see Table 1). In the treatment of depression, XYS has unique theoretical advantage and rich scientific connotation, as it is consistent with the overall concept and characteristics of TCM based on syndrome differentiation. Clinically, XYS is the most common TCM prescribed for the treatment of anxiety and depression caused by liver Qi stagnation, blood deficiency, and spleen deficiency (Zhou et al., 2011). XYS is often used as a single treatment or in combination with other western drugs in the treatment of depression. Clinical studies have shown that XYS has significant antidepressant effects without evident adverse reactions (Qin et al., 2010). This article provides an overview of existing clinical and experimental research, describing the pathogenesis of depression and potential therapeutic targets, while systematically reviewing the research progress of treating depression using XYS.

**TABLE 1:** Composition of XYS and Eight ingredients in XYS sample (XYS is derived from “Taiping Huimin Heji Jufang” and is composed of these eight Chinese medicines) (Ding et al., 2017a).

Medicinal plant	Amount(g)						
Radix Angelicae Sinensis	15						
Radix Paeoniae	15						
Radix Bupleuri	15						
Radix Glycyrrhizae	6						
Poria ((Poria cocos	15						
Rhizoma Atractylodis Macrocephalae	15						
Herba Menthae	6						
Rhizoma Zingiberis Recens	15						
**Eight ingredients in XYS sample**							
Ingredient	PubChem CID	Molecular Formula	Molecular Weight	Molecular structure	Separationtechnology	Characterization techniques	References
Palmitic acid	985	C16H32O2	256.42 g/mol	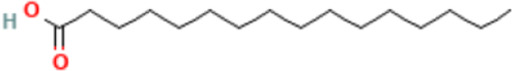	freeze drying, extraction separation	chromatography, spectrometry	Piovesana, Aita et al. (2021)
Curcumin	969516	C21H20O6	368.4 g/mol	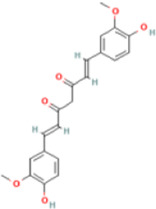	ultrasonic, pressurized liquid extraction, microwave, reflux	chromatography, spectrometry, Fourier transform infrared (FT-IR), Near-infrared (NIR), Raman, and ultraviolet/visible (UV-Vis)	Kotha and Luthria (2019)
Liquiritin	503737	C21H22O9	418.4 g/mol	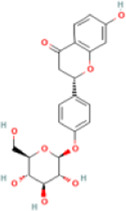	extraction, drying, centrifugal separation	Chromatography, Fourier transform infrared (FTIR), Electrospray ionization mass spectrometry (ESI-MS), 1H Nuclear Magnetic Resonance (NMR) and 13C NMR spectra	Wang, Shan et al. (2020)
Saikosaponin D	107793	C42H68O13	781 g/mol	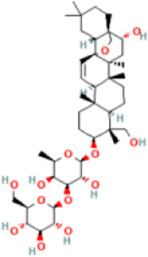	accelerated solvent extraction (ASE), centrifugal separation, drying, heat-reflux extraction, ultrasonic-assisted extraction, solvent-partitioning extraction	High performance liquid chromatography coupled with mass spectrometer (HPLC-MS), Liquid chromatography coupled with tandem mass spectrometry (LC-MS/MS)	Li, Li et al. (2018)
Paeoniflorin	442534	C23H28O11	480.5 g/mol	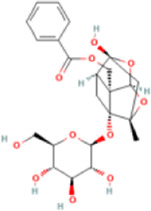	drying, extraction, chromatographic separation	NMR	Papandreou, Magiatis et al. (2002)
Saikosaponin B1	9,875,547	C42H68O13	781 g/mol	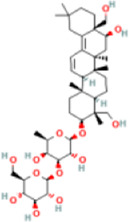	drying, filter separation	High Performance Liquid Chromatography (HPLC)	Ohtake, Nakai et al. (2004)
Atractylenolide II	14,448,070	C15H20O2	232.32 g/mol	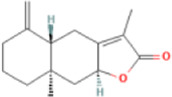	chromatographic separation, drying, extraction	HPLC	Zhang, Liu et al. (2017)
Pachymic acid	5,484,385	C33H52O5	528.8 g/mol	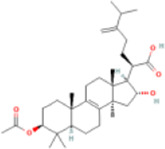	chromatographic separation, extraction	NMR, IR spectra	Zhou, Zhang et al. (2008)

## 2 Clinical applications of XYS for the treatment of depression

The use of XYS alone or in combination with western medicines has been found to relieve symptoms of depression with fewer side effects than traditional antidepressants, and it is thus considered a promising antidepressant for future use. A systematic review has shown that XYS and antidepressants have a significant comprehensive effect on the treatment of depression. The Hamilton depression scale (HAMD) and self-rating depression scale (SDS) scores of XYS were superior to those of antidepressants alone without significant side effects (Kou and Chen 2012; Zhang et al., 2012). Based on gas chromatography—mass spectrometry (GC-MS) metabolomics, Liu et al. found that the plasma metabolic profile of patients with depression was different when compared with a healthy control group, and that XYS significantly regulated depression symptoms by reversing metabolite and pathway levels to that of the control group (Liu X. et al., 2020). By recruiting 25 patients with depression and 33 healthy volunteers and using GC-MS urinary quantitative metabolomics, XYS was found to have a better priority for the treatment of depression (Tian et al., 2016). Chen et al. found that the Xiao Yao Pill containing Chaihu had a regulatory effect on the nervous and endocrine systems of patients with liver Qi stagnation and spleen deficiency syndrome (LSSDS), and helped to improve their clinical performance (Chen et al., 2005). A meta-analysis of ten randomized controlled trials involving 735 patients showed that when compared with antidepressants alone, XYS as an adjuvant therapy was beneficial in relieving depression symptoms. Furthermore, XYS in combination with antidepressants could improve the efficacy of the latter and reduce their HAMD scores and adverse reactions such as insomnia and constipation (Man et al., 2014). Another meta-analysis conducted for 607 patients with post-stroke depression (PSD) identified from seven trials revealed that using XYS as an adjuvant therapy was beneficial without adverse effects, reducing both the HAMD and Scandinavian stroke scale scores evidenced when using the antidepressants alone (Jin et al., 2018). A clinical study of 180 patients with functional dyspepsia (FD) associated with perimenopausal depression, found that Xiaoyao Pill (XYS) had positive therapeutic effects on the Hamilton depression rating scale (HRSD), gastrin, motilin levels, and gastric emptied rate of perimenopausal FD patients, and it could effectively improve related symptoms (Du et al., 2014). The TCM derived from Jiawei Xiaoyao (JWXY) also had positive effects when used as a main prescription (Su et al., 2019). For mild to moderate depression with anxiety symptoms, JWXY was as effective as sertraline in reducing depressive symptoms and it showed a faster onset and longer lasting effect than sertraline in the reduction of anxiety symptoms; it also improved sleep quality and physical anxiety symptoms. As JWXY is safe and cheaper than traditional antidepressants, it may be the preferred choice for treating depression with anxiety symptoms. Xiaoyaosan can effectively improve blood sugar level and depressive symptoms, regulate the body’s cortisol (Cor) and adrenocorticotropic hormone (ACTH) levels, and reduce the adverse reactions caused by antidepressant drugs in the treatment of the depressive disorder of type 2 diabetes with liver stagnation and spleen deficiency (Zhang J. et al., 2021). The clinical efficacy of Jiawei Xiaoyaosan combined with fluoxetine in the treatment of depression is good, and it can significantly improve the depression state of patients (Liu J. et al., 2020). The clinical efficacy of Danzhi Xiaoyaosan in the treatment of mild to moderate depression is significant, and its side effects are small. Part of its antidepressant mechanism may be achieved by regulating serum 5-HT, CORT, and BDNF (He 2019). With the progress of treatment, Xiaoyao Powder can restore the functions of the hypothalamic-pituitary-gonadal axis and the HPA (Jiang et al., 2015). Sixty patients with mild to moderate depression were randomly divided into two groups to observe the clinical efficacy of Xiaoyaosan. The results showed that Xiaoyaosan can significantly improve the clinical symptoms of mild to moderate depression without adverse reactions (Yang and Lin, 2015). Xiaoyaosan combined with sertraline can quickly and effectively enhance the clinical efficacy of sertraline and improve clinical symptoms, which is worthy of clinical promotion (Zhang, 2015). Some researchers have found that Xiaoyaosan has a definite effect in the treatment of depression and that the effect is fast: most patients return to normal after 6 weeks of treatment (Feng et al., 2014). Overall, these clinical studies have shown that XYS is effective for depression treatment and generally well-tolerated without obvious adverse reactions (see Table 2). It can be used safely when based on reasonable syndrome differentiation.

**TABLE 2:** The clinical application of XYS in the treatment of depression.

Characteristics of study											
Study ID	Population	Study design	Sample	Experiment group	Control group	Course (week)	Outcomes	administration pathway	Followup (month)	adverse reaction	relapses
Du, Ming et al. (2014)	functional dyspepsia (FD) associated with perimenopausal depression	RCT	Exp: 90								
			Con: 90	Xiaoyao pill	Placebo	8	HRSD, gastric emptying rate	Exp: 3g, bid	6	none	Exp: 0
								Con: 3g, bid			Con: 5
Chen, Ji et al. (2005)	Liver stagnation and spleen deficiency syndrome (LSSDS)	RCT	Exp: 41								
			Con: 17	Xiao Yao Wan	Zhi Bai Di Huang Wan	4	Self-rating anxiety scale and self-rating depression scale, β-endorphin, Epinephrine and Dopamine	Exp: 8 pills, tid	0	none	Exp: 0
								Con: 8 pills, tid			Con: 0
Su, Fan et al. (2019)	mild to moderate depression with anxiety symptoms	RCT	Exp: 105								
			Con: 105	Jiawei Xiaoyao (JWXY) capsule + sertraline placebo	Sertraline + JWXY placebo	8	HAMD, HAMA and the Clinical Global Impression Scale	Exp: Jiawei Xiaoyao capsule 10 g* 2/d + sertraline placebo	1	Exp: dry mouth, headache, sweating, nausea, dizziness	Exp: 14
										Con: sertraline 50 mg/d + Jiawei Xiaoyao placebo	Con: 21
Zhang et al. (2021b)	type 2 diabetes with comorbid depression of liver spleen deficiency type	RCT	Exp: 46								
			Con: 46	Xiaoyaosan + escitalopram oxalate tablets	escitalopram oxalate tablets	6	HAMD, SDS, FPG, 2 hPG, HbA1c, COR, ACTH	Exp: Xiaoyaosan (Granule, 1 dose/d) + Con			
								Con: 5–10mg, qd	0	none	Exp: 0
											Con: 0
Liu et al. (2020a)	depression	RCT	Exp: 40								
			Con: 40	Xiaoyaosan + fluoxetine	fluoxetine	8	HAMD, SDS	Exp: Xiaoyaosan (Decoction, 1 dose/d) + Con			
								Con: 20mg, bid	0	Exp: leukopenia, gastrointestinal reactions, dizziness, edema	Exp: 0
										Con: leukopenia, gastrointestinal reactions, dizziness, edema	Con: 0
He. (2019)	mild to moderate depression	RCT	Exp: 46								
			Con: 46	Danzhi Xiaoyaosan	Sertraline	8	HAMD, SDS, 5-HT, CORT, ACTH	Exp: Danzhi Xiaoyaosan (Decoction, 1 dose/d)			
								Con: 50mg, qd	0	Exp: gastrointestinal reactions	
										Con: gastrointestinal reactions	Exp: 0
											Con: 0
Jiang, Zhou et al. (2015)	depression	RCT	Exp: 44								
			Con: 44	Xiaoyaosan + citalopram	citalopram	6	HAMD, CORT, ACTH, T3, T4, TSH, E2	Exp: Xiaoyaosan (Decoction, 1 dose/d) + Con			
								Con: 20mg, qd	0	Exp: nausea, vomiting, tachycardia	
										Con: nausea, vomiting, tachycardia	Exp: 0
											Con: 0
Yang and Lin, (2015)	mild to moderate depression	RCT	Exp: 30								
			Con: 30	Xiaoyaosan	Flupentixol and Melitracen Tablets	4	HAMD	Exp: Xiaoyaosan (Decoction, 1 dose/d)			
								Con: 10mg, qd	0	none	Exp: 0
											Con: 0
Zhang. 2015	depression	RCT	Exp: 56								
			Con: 56	Xiaoyao pill + sertraline	sertraline	6	HAMD	Exp: Xiaoyao pill (8 pills, tid) + Con			
								Con: 50–100mg, qd/bid	0	none	Exp: 0
											Con: 0
Feng, Tian et al. (2014)	depression	single-arm clinical trials	Exp: 62	Xiaoyaosan	-	8	HAMD, CGI, TCM syndrome scale	Exp: Xiaoyaosan (Decoction, 1 dose/d)	0	Exp: dry mouth, thirst, nausea	Exp: 0
											Con: 0

## 3 Pharmacological mechanisms of XYS for the treatment of depression

### 3.1 Neurotransmitters and their receptors

**FIGURE 1:**
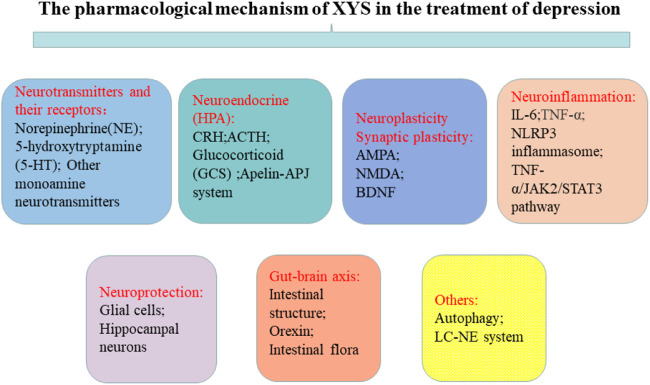
Potential therapeutic targets based on the pathogenesis of depression. The treatment goals of depression include neurotransmitters and their receptors, neuroendocrine (HPA), neuroplasticity synaptic plasticity, neuroinflammation, neuroprotection, and the gut-brain axis.

**FIGURE 2:**
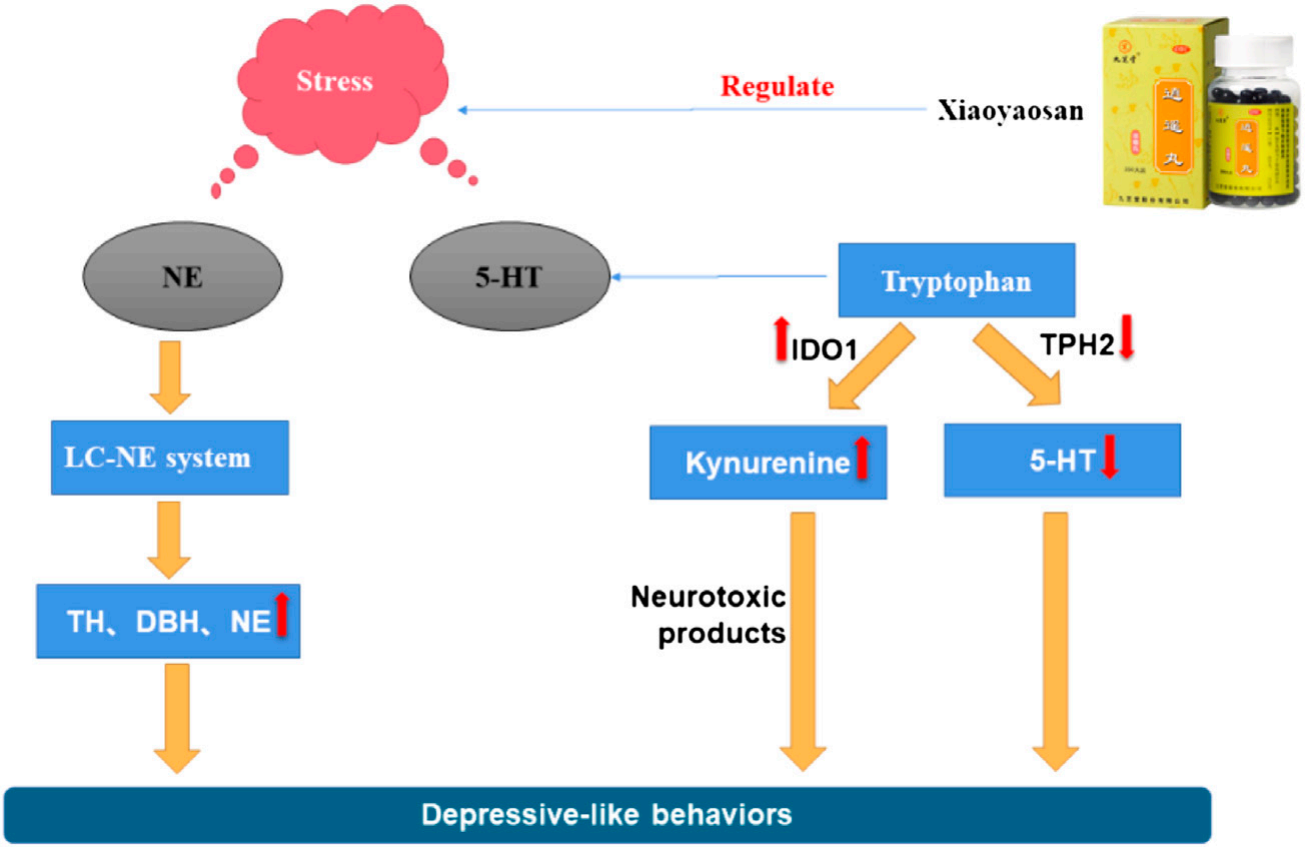
Mechanism of the stress-induced changes in NE and 5-HT and the regulating effect of Xiaoyaosan.

Figure 1 one of the current hypotheses for the pathogenesis of depression is the monoamine neurotransmitter hypothesis. Monoamine neurotransmitters, also known as biological amine neurotransmitters, have a wide range of biological activities, and are important for functions such as the regulation of body temperature, emotional responses, behavioral state, and mental activities. The monoamine neurotransmitter hypothesis suggests that the loss or decrease of NE, 5-HT, or other monoamine neurotransmitters in the central and peripheral regions may induce depression (Carpenter et al., 2004; Castrén 2005). Zhang et al. (Zhang et al., 2018) found that a Jiawei Xiaoyao (XYS) capsule promoted the increase of cortisol levels and expression of tyrosine hydroxylase, and improved the expression of monoamine neurotransmitters (including 5-HT and NE). The modified Xiaoyao capsule could also save the depressive phenotype and cognitive behavior of zebrafish by changing the levels of endogenous cortisol and monoamine neurotransmitters. Dysregulation of monoamine neurotransmitters and their receptors, especially 5-HT, may thus be the basic cause of depression. Studies have found that XYS up-regulated the 5-HT content in the cerebral cortex of the rat depression model induced by chronic restraint stress (CRS) (Bao et al., 2008), and increased the 5-HT content in the hippocampus of rat with postpartum depression (Wang and Qin 2010). Activation of the 5-HT1A receptor has been shown to increase the release of DA. Yin et al. found that XYS/room temperature super-extraction systems (RTSES) could significantly increase the shuttle activity of C57BL/6J mouse and reduce the resting time on the forced swimming test (FST) and tail suspension test (TST), as well as increase insulin sensitivity in mice with anxiety and depression caused by reserpine. The insulin sensitivity of mice also confirmed that the activation of the brain 5-HT1A receptor led to the anxiolytic and antidepressant effects of the XYS/RTSES treatment (Yin et al., 2012). XYS may thus be a modulator of monoamine neurotransmitters (Kong et al., 2010). 5-HT comes from tryptophan, tryptophan generates 5-hydroxytryptophan (5-HTP) under the action of tryptophan hydroxylase, and then decarboxylates under the catalysis of 5-hydroxytryptophan decarboxylase to generate 5-HT. Therefore, the level of tryptophan affects the amount of 5-HT in the brain. In order to further clarify the mechanism of the decline of monoamine neurotransmitters, some studies have found that the activity of indoleamine 2,3-dioxygenase (IDO) in the serum of patients with depression is significantly increased, and the rate of tryptophan decomposition is accelerated. This inhibits the metabolism of tryptophan to the 5-HT pathway and reduces the concentration of the neurotransmitter 5-HT in the synaptic cleft, thereby accelerating the occurrence of depression. Stress-induced stimulation of indoleamine 2, 3-dioxygenase 1 (IDO1) can affect the metabolic conversion of tryptophan to kynurenine and thus reduce the ability of tryptophan hydroxylase (TPH) to synthesize 5-HT. XYS can reduce the number of microglial cells and the expression of IDO1 in the dorsal fissure nucleus of depressed mice, and inhibit 5-HT synthesis (Wang M. et al., 2018). As tryptophan is the precursor of 5-HT, microorganisms can activate IDO1 to deplete tryptophan through the kynurenine pathway, resulting in the decrease of 5-HT, which leads to depression. Jiao et al. found that XYS could improve the metabolism of tryptophan by adjusting the expression levels of TPH2 and IDO1 in a rat depression model established using the chronic fixed stress (CFS) method, thereby exerting an antidepressant effect (Jiao et al., 2019). Ding et al. (Ding et al., 2014) found that after exposure to continuous chronic stress, the activated locus coeruleus (LC)-NE system had a significant impact on the occurrence and development of depression in rat. After XYS treatment, the expression of NE, thyroid hormone (TH), and adrenocorticotropin-releasing factor (CRF) in rat was significantly reduced when compared with the control group. Zhang et al. found that XYS treatment affected the levels of DA, 3,4-dihydroxyphenylacetic acid (DOPAC), and homovanillic acid (HVA) in the various brain regions of chronic unpredictable mild stress (CUMS) rat model; levels were significantly reduced in the prefrontal cortex and the nucleus accumbens, along with DA receptor 1 (D1R) mRNA and protein expression. The specific mechanism of XYS may be related to the free and unrestricted dopamine (DA) scattered in the central nervous system and to the increased expression of its receptors and transporters (Zhang H. et al., 2021). Guo et al. found that XYS may play an anti-depressive role by increasing progesterone and allopregnanolone and decreasing pregnenolone (PREG) in the hippocampus and amygdala of the CUMS-stimulated rat model, thereby enhancing the release of γ-aminobutyric acid (GABA) and N-methyl-D-aspartate (NMDA) receptors, inhibiting the release of glutamate receptors, and reducing the inhibitory effects of GABA (Guo et al., 2017). These results indicate that XYS can effectively improve the depression-like behavior of rat by inhibiting the activity of LC-NE neurons. In summary, the antidepressant mechanism of XYS has been shown to be related to monoamine neurotransmitters (see Figure 2).

### 3.2 Neuroendocrine (HPA) axis

**FIGURE 3:**
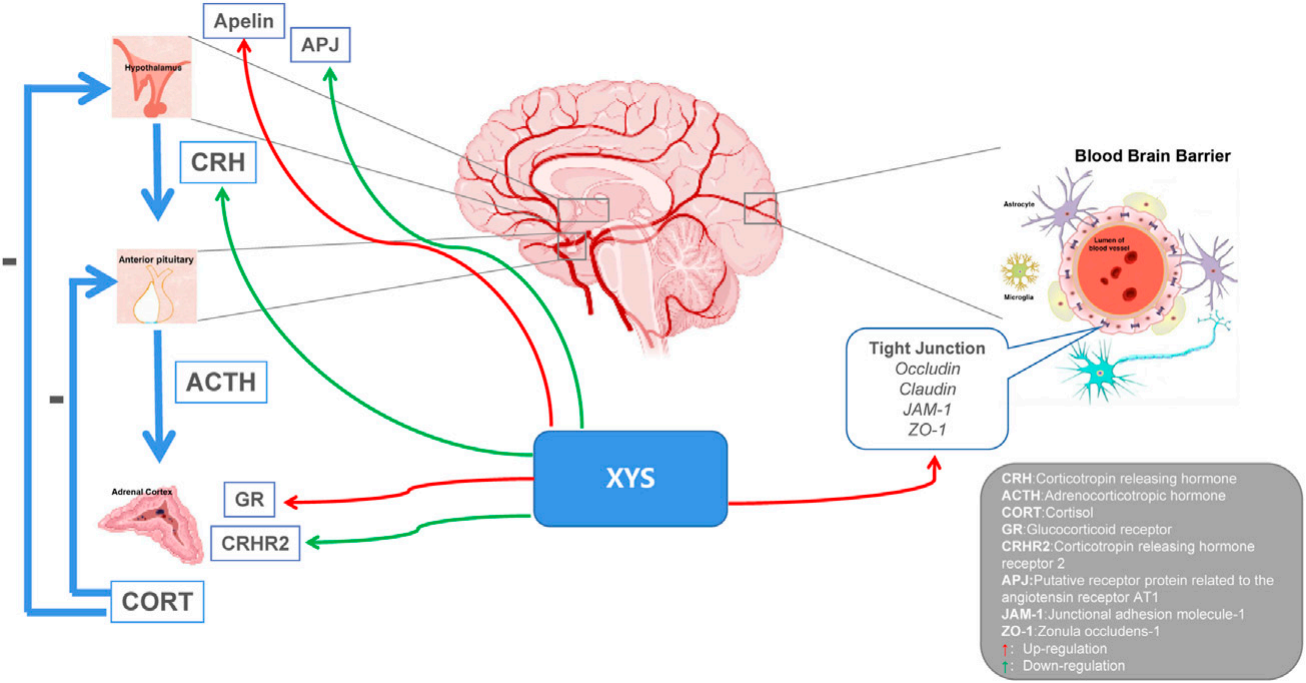
Potential relationship between Xiaoyaosan, depression and HPA axis. Xiaoyaosan can up-regulate the level of apelin in the hypothalamus and down-regulate the level of APJ in the hypothalamus. Down-regulation of CRH and CRHR2 levels, up-regulation of GR expression, and regulation of HPA axis hyperactivity. Elevate the expression of tight junction-related genes and reverse the damage of glucocorticoids to the permeability of the blood-brain barrier. Red arrows indicate upregulation. Green arrows indicate downregulation.

Depression is categorized as a mental disorder, and neuroendocrine experiments have found that endocrine hormones are closely related to behavior and mood. From the perspective of neurobiology, the occurrence of depression is closely related to the dysfunction of the HPA axis (Cai et al., 2015). When the human brain is stimulated by stress, the cerebral cortex is affected, the hypothalamus sends out signals, HPA axis participates in neuroendocrine regulation, and secretion of corticotropin releasing factor (CRF) increases. Excessive CRF is transferred from the portal system to the pituitary gland to synthesize adrenocorticotropic hormone (ACTH), which circulates throughout the body stimulating the adrenal cortex to produce cortisol (Olloquequi et al., 2018). The mechanism of the hyperactivity of the HPA axis is the excessive secretion of CRF. CRF is a key factor in regulating the stress response of the body and is related to various mental diseases such as depression. In addition, cortisol content in depression patients is closely related to the clinical manifestations of disease and cortisol has a key role in the pathogenesis of depression. XYS has been shown to regulate HPA axis disorders in the depression rat model at multiple targets and levels and to increase the expression and accelerate the transport of central glucocorticoid receptors in depressed rat (Chen et al., 2008b). Lu et al. found that XYS could promote the expression of glucocorticoid receptors and restore the negative feedback regulation of the HPA axis, thereby relieving depression (Lu et al., 2018). Wu et al. found that Danzhi XYS could inhibit overactivity of the HPA axis, regulate monoamines and amino acid neurotransmitters in the hippocampus, and improve the depression-like behavior of rat under chronic stress (Wu et al., 2016). Zhu et al. found that XYS could improve depression-like behavior by down-regulating the level of corticotropin-releasing hormone (CRH) receptor 2 in social isolation and CUMS models, and improve HPA axis overactivation (Zhu et al., 2014). The apelin/APJ system is considered to play an important role in the function of the HPA axis. Yan et al. found that XYS could improve depressive behavior by up-regulating hypothalamic apelin levels and down-regulating hypothalamic APJ levels in a CUMS rat model. Thus, changes in the hypothalamic apelin/APJ system may be a depression target, and XYS may have a regulatory effect on it (Yan et al., 2018). Chronic stress can destroy the permeability of the blood-brain barrier (BBB) (Duffy et al., 2014). Glucocorticoids (GCs) are important hormones in the human body, and their secretion is mainly regulated by the HPA axis. Yu et al. found that XYS can partially reverse the inhibition of GCs-induced cell proliferation and invasion promoting the expression of tight junction-related genes; this allows reversing the damage to the BBB permeability induced by chronic stress (Yu et al., 2020). Despite these breakthroughs, the exact mechanism by which XYS restores the HPA axis still needs to be clarified (see Figure 3).

### 3.3 Neuroplasticity and synaptic plasticity

Synaptic plasticity is an important part of learning and memory from the perspective of neurobiology. It indicates that the structure and function of synapses can change under the influence of the continuous activity of neurons, with adjustable characteristics. Synapses are thus crucial for neuronal networks and information transmission (Kim et al., 2006). Furthermore, when synaptic plasticity is reduced and neurons are functionally impaired, learning and memory are also impaired (Coleman et al., 2004), which is associated with a variety of neuropsychiatric diseases (Südhof, 2008; Gilman et al., 2011). In recent years, an increasing number of studies has shown there is a close relationship between synaptic plasticity and depression, and improving synaptic plasticity can help reversing depressive symptoms and the associated cognitive decline (Sullivan et al., 2000). Synapses are crucial for the transmission and processing of information between neurons, and reductions in the number of synapses or structural damage to neurons will affect the release of neurotransmitters, resulting in information transmission disorders in the central nervous system. Using electron microscopy, Liang et al. found that the pyramidal cells in the hippocampal CA1 region of the chronic stress rat model had several ultrastructural damages to the nucleus, mitochondria, and other important organelles and synapses, while the XYS group had no evident abnormalities in the CA1 region (Liang et al., 2009). Ao et al. found that polyphasic stress could damage synaptic structures and affect their connections in rat, whereas XYS could reduce the stress damage to the original synapses and synaptic connections, promote the formation of new synapses and synaptic connections, and reduce learning and memory impairment (Ao et al., 2006).

#### 3.3.1 α-Amino-3-hydroxy-5-methyl-4-isoxazolepropionic acid (AMPA) receptors

Numerous studies have shown that the pathogenesis of depression may be closely related to a disorder in the glutamatergic system (Henter et al., 2018; Moriguchi et al., 2019). Glutamate is the main excitatory neurotransmitter of the central nervous system, and it plays an important role in the differentiation, development, and growth of neurons. Glutamate is also involved in synaptic signal transmission and learning and memory processes, and it plays a key role in the maintenance of synaptic stability and plasticity (McDonald and Johnston 1990). Studies have shown that AMPA receptors, as ionotropic glutamate receptors, are important in both synaptic plasticity and cell death caused by neurological disease and dysfunction (Whitehead et al., 2017). AMPA receptors can participate in the pathogenesis of depression by changing synaptic plasticity, and directly or indirectly participate in the mechanism of depression as antidepressants (Aleksandrova et al., 2017; Zhou et al., 2019). Liang et al. found that chronic immobilization stress (CIS) induced a decrease in GluR2 mRNA and an increase in GluR1 mRNA in the hippocampal CA1 area of rats; ultrastructural damage was also observed in this region. However, XYS could reverse this trend, indicating it can produce a certain antidepressant effect, and suggesting it is related to the hippocampal synaptic plasticity of AMPA receptors (Liang et al., 2013). The PDZ domain of 95 kDa postsynaptic density protein (PSD-95) regulates the function of AMPA receptors through protein-protein interactions. PSD-95 can combine with molecules in the NMDA receptor signaling pathway to form a signaling complex. Meng et al. found that XYS can reverse the slow weight gain and related symptoms of LSSDS in rat exposed to chronic immobilization stress (CIS), and up-regulate the levels of the hippocampal PSD-95 and synaptophysin, thereby improving learning and memory impairment and synaptic plasticity (Meng et al., 2013). Xi et al. found that XYS (FWP) could reverse the decrease of GluR1 and GluR2/3 in hippocampal region CA3, p-GluR1 in hippocampal region CA3, and p-GluR2 in hippocampal regions CA1 and CA3 induced by CRS in rat models and inhibit the increase of GluR1 in hippocampal region CA1 and DG, p-GluR1 in hippocampal region CA1, and p-GluR2 and GluR3 in amygdala BLA. It is suggested that FWP may play an antidepressant role by regulating AMPA-type glutamate receptor homeostasis in the amygdala and hippocampus to relieve the symptoms of liver depression and Malcan deficiency syndrome (Xi et al., 2020).

Glutamate concentration and action time beyond the physiological range will lead to the production of excitatory neurotoxicity damage to neurons. This is one of the pathological mechanisms of social stress disorders, such as affective disorder. This mechanism is associated with damage to astrocytes (AS) and excitatory amino acid transporters (EAATs) due to long-term stress. Zhou et al. found that XYS exerted antidepressant effects through the NR2B and PI3K/Akt signaling pathways to alleviate hippocampal neuronal damage induced by glutamate-mediated excitotoxicity in rat (Zhou et al., 2021). Liu et al. found that XYS could reverse the expression of glial fibrillary acidic protein (GFAP), EAAT1, EAAT2, and Neuronal nuclei antigen (NeuN) in the prefrontal cortex of CUMS mice, and increase the content of glutamate, thereby exerting antidepressant effects (Liu et al., 2019). Ding et al. found that XYS could regulate the depression-like behavior of mice induced by CUMS, which might be related to changes in the hippocampal glutamate/glutamine cycle and glutamate transporter (GLT) 1, suggesting that the antidepressant effect of XYS is related to the glutamate energy system (Ding et al., 2017a). Chen et al. found that XYS may regulate glutamine and glutamate metabolism in rat to maintain the ammonia nitrogen balance and promote energy metabolism, thereby improving depression-like behavior and liver injury symptoms induced by CUMS (Chen et al., 2020a). Ding et al. found that chronic administration of XYS may normalize the expression of the glial fibrillary acidic protein (GFAP) in the hippocampus of mice with CUMS, thereby exerting an antidepressant effect (Ding et al., 2017b).

#### 3.3.2 NMDA receptors

The dysfunction of N-methyl-D-aspartic acid receptor (NMDA) receptors is related to certain neuropsychiatric disorders (Zoicas and Kornhuber 2019), and there is substantial evidence supporting NMDA receptor dysfunction in patients with depression (Balu 2016). Song et al. found that XYS could improve the body weight and food intake of rat exposed to CUMS, affect the activity of AS, reverse the changes of corticosterone in the HPA axis, and downregulate the level of NMDA receptors in the hippocampus (Song et al., 2020).

#### 3.3.3 BDNF

**FIGURE 4:**
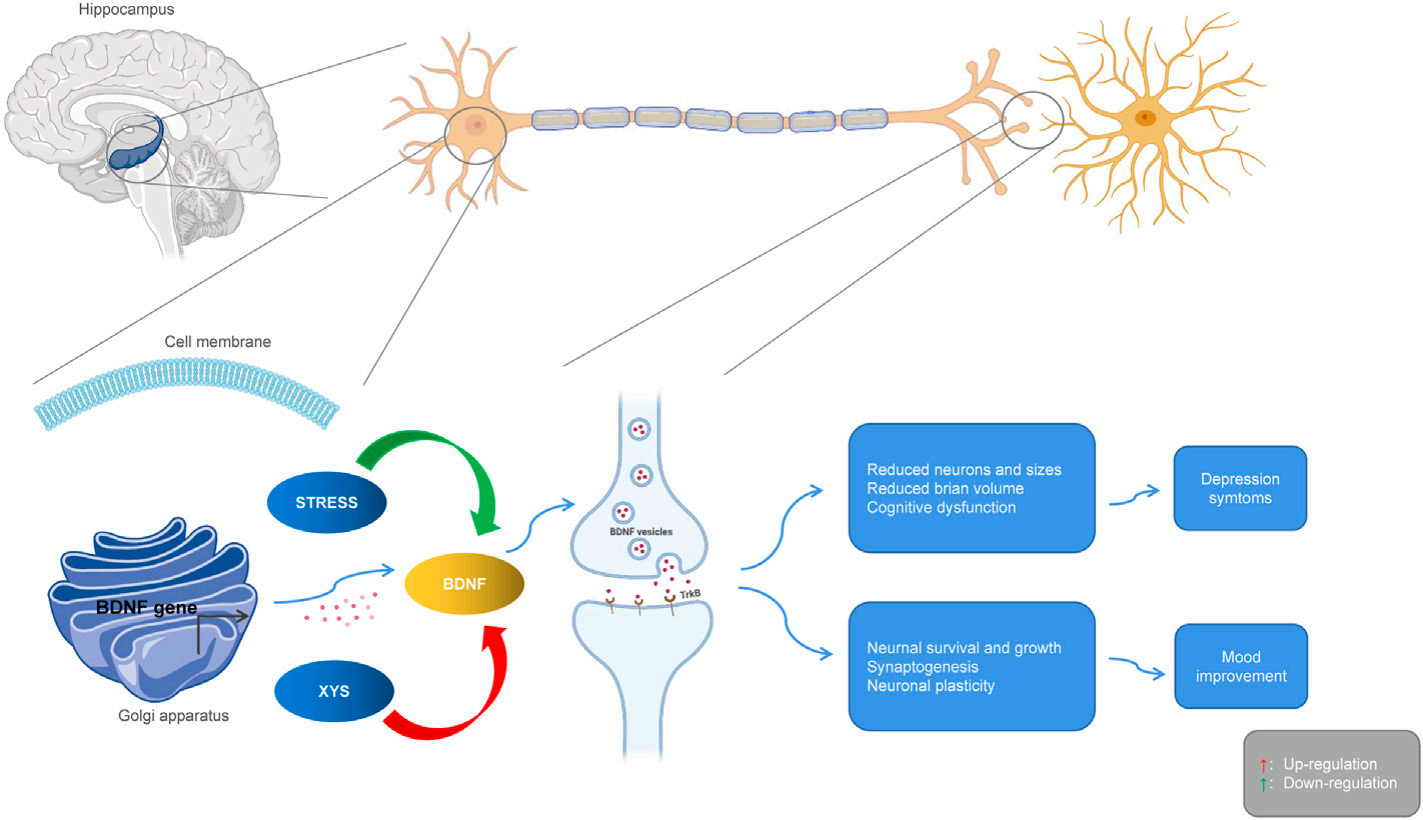
Interactions between Xiaoyaosan, depression and BDNF. Stress downregulates BDNF, leading to atrophy and loss of hippocampal neurons, producing depressive symptoms. Xiaoyaosan up-regulates BDNF, promotes neuron survival and synaptogenesis, and improves depressive mood. Red arrows indicate upregulation. Green arrows indicate downregulation.

The expression of neurotrophic factors in the serum and brain regions of patients with depression are significantly different from those of healthy people, suggesting that neurotrophic factors may become new markers for depression. BDNF is the most abundant neurotrophic protein in the brain tissue, and it is crucial for the growth, proliferation, survival, and synaptic activity of neurons (Machaalani and Chen 2018). BDNF protein and mRNA expression can be detected in various parts of the cerebral cortex, hippocampus, olfactory bulb, basal forebrain, midbrain, and hypothalamus (Bathina and Das 2015). Due to its role in regulating the synaptic structure and neuroplasticity, BDNF has attracted extensive attention in the pathogenesis of stress disorders. As a key molecule in central nervous system cascade signaling, BDNF is closely related to the occurrence of depression, and it is an important target of antidepressant therapies. Evidence from clinical studies has shown that part of the pathophysiology of depression is the down-regulation of BDNF and its tyrosine receptor kinase B (TrkB), which in turn leads to neuron atrophy and loss in different brain regions, including the hippocampus. Studies have found that XYS (Ding et al., 2017b) could prevent depression-like behaviors by increasing the expression of BDNF. After treatment with XYS for 28 days, BDNF gene expression in the depressed rat model was up-regulated while the expression of BDNF and cyclic AMP response element binding (CREB) proteins was significantly increased (Wang J. et al., 2018). Xiaoyao Jieyu Powder (XYS) was found to improve post-stroke depression in rat by regulating BDNF, the cannabinoid receptor, and CRF in the ventral tegmental area of the midbrain (Wang C. et al., 2019). Studies have also confirmed that XYS has anti-anxiety and anti-depressive effects (Zhi-wei et al., 2004; Chen et al., 2008a), as well as a beneficial effects on stressed rat. The BDNF of the frontal cortex and hippocampal CA1 area of rat exposed to CIS decreased, while the levels of TrkB and neurotrophin 3 (NT3) increased, but XYS could reverse this trend to a large extent. Curcumin is one of the main components of XYS. Studies have found that it can increase hippocampal neurogenesis in chronically stressed rat and prevent the stress-induced reduction of 5-HT1A mRNA and BDNF protein levels in hippocampal subregions. These two molecules are involved in hippocampal neurogenesis. Moreover, curcumin can reverse or protect hippocampal neurons from chronic stress induced damage by up-regulating the 5-HT1A receptor and BDNF, which may be the basis of antidepressant effect of curcumin (Xu et al., 2007). In addition, XYS promoted lipopolysaccharide-induced hippocampal neurogenesis by increasing BDNF, nerve growth factor (NGF), TrkB, TrkA, and CREB (Fang et al., 2020). Modified XYS (MXYS) significantly improved body weight and depression-like behavior, up-regulated BDNF level, and restored hippocampal neurogenesis in CUMS exposed mice (Gao et al., 2018). The neurotrophic theory, as the central theory for depression, provides a good explanation for the pathogenesis of depression with some shortcomings. BDNF and TrkB, as the most important members of the neurotrophic factor family, are thought to be closely related to depression, and often work together. Therefore, the antidepressant effects of XYS may be carried out via the BDNF/TrkB signaling pathway (see Figure 4).

### 3.4 Neuroinflammation

**FIGURE 5:**
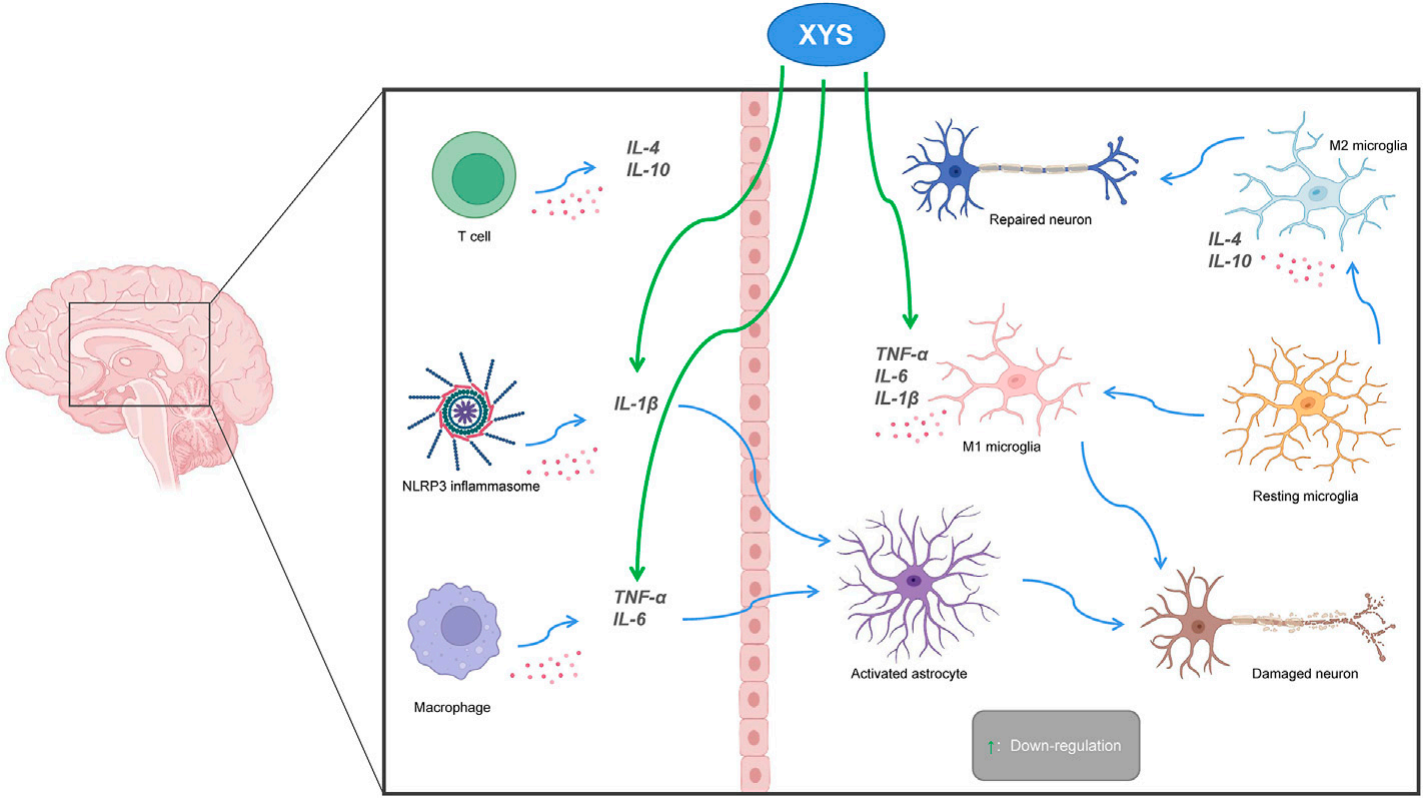
The relationship between Xiaoyaosan, depression and neuroinflammation. Pro-inflammatory cytokines (IL 1β, IL6, TNF-α) activate astrocytes, leading to neuronal destruction. Down-regulation of pro-inflammatory cytokines by Xiaoyaosan inhibits activation of astrocytes and avoids neuronal damage. M2 microglia produce anti-inflammatory cytokines (IL4, IL10) that promote neuronal repair. Green arrows indicate downregulation.

Recent studies have found that neuroinflammation is associated with the occurrence of depression, and that NLRP3 inflammasomes, cytokines, astrocytes, and microglia are all involved. NLRP3 is a regulator of the inflammatory response and can regulate the expression of IL-1β. Microglia are activated during infection or stress, leading to elevated levels of inflammatory factors in the brain that disrupt neuronal structure and function. Astrocytes sense the inflammatory signals activated by microglia. Activated astrocytes are divided into M1 type and M2 type. Those of the M1 type release a variety of pro-inflammatory factors and have neurotoxic effects. Those of the M2 type can up-regulate neurotrophic factors, promote the release of anti-inflammatory factors, and have neuroprotective effects. The secretion of pro-inflammatory cytokines such as IL-1β, IL-6, and TNF-α is increased in patients with depression, while the secretion of anti-inflammatory cytokines such as IL-4 and IL-10, among others, is decreased (Howren et al., 2009; Dowlati et al., 2010). Animal experiments have shown that the lateral ventricular injection of lipopolysaccharides (LPSs) can induce a neuroinflammatory state in the hippocampus and increase the mRNA expression of pro-inflammatory cytokines IL-6 and TNF-α (Tang et al., 2016), which leads to depression-like behavior. Consequently, the occurrence of depression is associated with inflammation. Chen et al. found that XYS could improve depression-like behaviors by regulating the NLRP3 inflammasome in the cerebral cortex of rat exposed to CIS (Chen et al., 2020b). The mitogen-activated protein kinase (MAPK) signaling pathway is involved in a variety of pathological processes. The c-Jun N-terminal kinase (JNK) is part of this pathway, and it plays an important role in apoptosis and neurological diseases. Li et al. suggested that XYS could alleviate hippocampal neuronal injury and reverse the effects measured in the hypertension labyrinth test, through the activation of the TNF-α/Janus kinase 2 (JAK2)/Signal transducer and activator of transcription 3 (STAT3) pathway in a rat model of CIS-induced anxiety (Li et al., 2017). Fang et al. found that Xiaoyao Pills (XYS) could improve the inflammatory response and nerve damage of hippocampal neurons induced by LPSs, as well as down-regulate the levels of inflammation-related cytokines and mediators, thereby exerting neuroprotective effects (Fang et al., 2020). After treatment with XYS, mouse body weight, behavior (as measured in novel, inhibition of feeding, open field, and maze tests), and hippocampal JNK, phosphorylated JNK (pJNK) and phosphorylated c-Jun (pcJun) levels changed, indicating XYS may act in the JNK signaling pathway for chronic stress in a mice depression model (Zhao et al., 2017; Zhao et al., 2020) (see Figure 5).

### 3.5 Neuroprotection

Yuan et al. found the antidepressant XYS mechanisms were related to neuroprotection using system-network pharmacological analysis (Yuan et al., 2020). Yin et al. found that in reserpine-induced anxiety and depression models, XYS/RTSE improved blood glucose regulation, increased insulin sensitivity, and had a neuroprotective effect on the glial cell injury system (Yin et al., 2012). Li et al. found that XYS reversed the damage observed in the hippocampal structure and function of rat exposed to CIS for 21 days, and had the ability to enhance immunity and stress resistance, promoting the regeneration of nerve cells (Li et al., 2019). Meng et al. found that the XYS decoction may help reducing hippocampal neuron apoptosis induced by oxidative stress, suggesting that the antidepressant effects of XYS may be caused by its protective effects on hippocampal neurons (Meng et al., 2012). Liu et al. found that XYS could protect the hippocampal neurons of ovariectomized rat and restore hippocampal E2 levels, thereby improving cognitive ability (Liu L. et al., 2020).

### 3.6 Brain-gut axis

The brain-gut axis is a two-way information communication system that integrates the neural, immune, endocrine, and metabolic pathways between the brain and the gastrointestinal tract (GI) (Appleton 2018). The information acquired by exogenous factors such as vision and hearing and endogenous factors such as thinking and emotion is transmitted from the brain to the GI, which then regulates its movements and secretions. Stimulation of the GI can also affect the activity of the central nervous system through the brain-gut axis. Recent studies have shown that functional disorders in the brain-gut microbiota axis plays an important role in the pathogenesis and progression of depression (Chen et al., 2018). Patients with depression have intestinal microbiota disorders, which are manifested as declines in the diversity and richness of the gut microbiota (Aizawa et al., 2016). In fact, regulating of the intestinal microbiota has become a hot topic in neuroscience and psychology, particularly how this affects psychological diseases such as depression and anxiety. Therefore, maintaining the balance of intestinal microbiota to adjust and affect the brain function may be an intervention method of great significance for the prevention and treatment of depression.

#### 3.6.1 Intestinal structure

In rodent models, psychological stimuli and stress increase gut permeability and promote bacteria to the systemic circulation and brain, thereby increasing the underlying incidence of depression. The expression of C-type natriuretic peptide (CNP) and natriuretic peptide receptor (NPR)-B in depressed rat decreased after XYS treatment, suggesting XYS may improve the symptoms of depressive gastrointestinal dysfunction by down-regulating the CNP signaling pathway (Li et al., 2015). Ding et al. found that XYS alleviated depression-like behaviors in rat with CUMS, effectively reversed the pathological and ultrastructural changes in the rat’s colon, thereby restoring intestinal permeability, and increased the level of serotonin in the hypothalamus and colonic mucosa (Ding et al., 2020). These results suggested XYS may improve the intestinal barrier function through the brain-gut axis and thus exert an antidepressant effect.

#### 3.6.2 Orexin, neuropeptide Y (NPY), and proopiomelanocortin (POMC)

Increasing evidence has shown that the orexin system is related to the pathogenesis of depression, and that its regulation is closely related to the occurrence and development of mental diseases. Hou et al. found XYS could increase the expression of orexin A/Oxidation resistance 1 (OXR1) in the lateral hypothalamus of CIS-induced depression rat model, indicating XYS could improve depression symptoms by regulating orexin A/OXR1 (Hou et al., 2020). Ma et al. found that XYS improved anorexia symptoms and depression-like behaviors in rat under CUMS by regulating the hypothalamic nesfatin 1 (NES1)-oxytocin (OT)-POMC neural pathway (Ma et al., 2019). Wang et al. found that a XYS decoction could regulate the expression of the leptin receptor (OB-R) and NPY in the hypothalamus of rat under CIS, while relieving discomfort symptoms such as loss of appetite and weight (Wang et al., 2012).

#### 3.6.3 Intestinal microbiota

Patients with depression show symptoms of psychological disorders which are often accompanied by symptoms of gastrointestinal dysfunction, such as functional dyspepsia and irritable bowel syndrome (Xie et al., 2013). They can also have lack of appetite, and variations in body weight are likely to be closely related to maladjustments in the intestinal microbiota. Recent studies indicate that botanicals and active ingredients, including XYS, improve depression-like behaviors by modulating GI microbiota. Furthermore, XYS may have an antidepressant role by regulating intestinal microbiota and its metabolites such as short-chain fatty acids (Zhu et al., 2019). Qiu et al. found XYS could regulate intestinal microbiota dysbiosis in rat with functional dyspepsia and LSSD exposed to CUMS (Qiu et al., 2017). Hao et al. found XYS improved the depressive and anxious behaviors of antibiotic-induced microbiome-depleted (AIMD) mice; the anti-depressant and anti-anxiety effects of XYS may be exerted via intestinal microbiota regulation and inhibition of the moderate activation of NLRP3 inflammasomes in the colon (Hao et al., 2021). Zhang et al. found that XYS could effectively improve the progression of colorectal cancer in mice exposed to a Chronic restraint stress (CRS) model, while protecting the integrity of the intestinal barrier, with some regulatory effects of the intestinal microbiota (Zhang et al., 2020).

### 3.7 Others

Autophagy is a lysosomal-dependent protein degradation pathway, which maintains the homeostasis of the intracellular environment through the degradation and recycling of damaged organelles and long-lived proteins; however, excessive autophagy is damaging (Klionsky et al., 2016). Previous studies have shown that in the process of depression, there is obvious autophagy activation, which leads to decreased survival rates for neurons and glial cells and to neuronal apoptosis (Zschocke et al., 2011). Wang et al. found that mice under CUMS and isolation exhibited depression-like behaviors *in vivo* and exhibited mixed apoptotic/autophagy phenotypes in the hippocampus; modified XYS alleviated neuronal apoptosis by regulating autophagy (Wang M. et al., 2019). Chronic stress induces an increase of NE transporter (NET) in the locus coeruleus, and disorders in the NE system may be one of the most important causes of depression (Chen et al., 2012). Ding et al. found that after exposure to persistent chronic stress, the activated LC-NE system had a significant effect on the occurrence and development of depression in rat (Ding et al., 2014). After Xiaoyao powder (XYS) treatment, the expression of NE, thyroid hormone (TH), and CRF decreased significantly in the experimental group when compared with the control group. These results indicated XYS can effectively improve depression-like behaviors in rat by inhibiting the activity of LC-NE neurons.

## 4 Conclusion and future directions

Depression presents an ongoing challenge for modern medicine as the pathogenesis of this disease is not fully understood, and there is no definite treatment method that can successfully stop or reverse depression. XYS is an important and valuable traditional Chinese medicine with a high level of safety and efficacy, and it could be used more widely for the treatment of depression in the future. Previous research into the mechanisms of the effects of XYS have been too broad. In the future, rather than only measuring the observed effects of XYS treatment using commercial products, in-depth research on specific genes or pathways with solid experimental basis should be conducted. It is also necessary to examine the existing hypotheses of depression using gene chip technology to detect whole genome changes and clarify the expression trends of each depression-related gene. Furthermore, there is also a lack of high quality standardized clinical studies that include a large number of patients and multiple centers and are performed as high-quality, randomized, double-blind controlled trials to verify the clinical efficacy of XYS for the treatment of depression. The occurrence of disease involves many aspects of body function, and the study of a single pathogenic factor cannot fundamentally clarify the nature of depression. The metabolic transformation of XYS components after entering the human body must also be considered in future investigations. In future studies, we must comprehensively apply various research techniques and methods to clarify the mechanisms of the action of the XYS antidepressant, and further explain these mechanisms using bioinformatics, network pharmacology, and metabolomics, to provide a basis for the research and development of TCM antidepressant drugs.
